# Comparison of adjuvant treatment regimens for high-risk hepatocellular carcinoma: a Bayesian network meta analysis and systematic review

**DOI:** 10.3389/fimmu.2024.1487353

**Published:** 2024-11-11

**Authors:** Jiahao Li, Yingnan Liu, Yuqi Qiu, Chao Qu, Jiarui Li

**Affiliations:** ^1^ Department of Interventional Therapy, The First Hospital of Jilin University, Changchun, Jilin, China; ^2^ Department of Radiology, The First Hospital of Jilin University, Changchun, Jilin, China

**Keywords:** hepatocellular carcinoma, TACE, HAIC, adjuvant therapy, immunotherapy, network meta-analysis

## Abstract

**Background:**

The five-year recurrence rate for patients with hepatocellular carcinoma (HCC) is as high as 70%. Patients with high-risk recurrence factors experience significantly poorer prognosis. Local regional therapies, including transarterial chemoembolisation (TACE), hepatic arterial infusion chemotherapy (HAIC), radiotherapy, and emerging immunotherapy, are commonly used adjuvant treatment options. We conducted an indirect comparison of these adjuvant therapies for such patients.

**Methods:**

We conducted a systematic search in public databases for relevant studies and assessed the efficacy and safety of the corresponding therapies by consolidating disease-free survival (DFS), overall survival (OS), and adverse events (AEs).

**Results:**

A total of eight randomised controlled trials were ultimately included. The Gelman-Rubin plot and kernel density estimation indicate that the stability of the combined model is satisfactory.

**Conclusion:**

immunotherapy is not inferior to local regional therapies in delaying tumour recurrence, however, the higher incidence of AEs remains a significant concern. Adjuvant radiotherapy demonstrated superior efficacy in delaying tumour recurrence compared to adjuvant TACE, although further support from phase III clinical trial evidence is required.

**Systematic review registration:**

https://www.crd.york.ac.uk/prospero/, identifier CRD42024576316.

## 1 Introduction

Hepatocellular carcinoma (HCC) is one of the top three causes of cancer mortality in 46 countries and ranks among the top five causes of cancer death in 90 countries globally. It is projected that by 2024, the incidence and mortality of liver cancer will increase by over 50% (1). To date, radical surgery remains the primary curative treatment for patients with early-stage tumours; however, its five-year recurrence rate still reaches 40% to 70% (2). Patients with high-risk recurrence factors, including vascular invasion, tumour diameter ≥5 cm, multifocal tumours, satellite nodules, poorly differentiated tumours (corresponding to Edmonson-Steiner grades III-IV), and non-capsulated tumours, exhibit significantly higher early and late recurrence rates, which pose a substantial challenge to patient prognosis (3–5). Therefore, it is essential to develop appropriate adjuvant therapies to reduce the postoperative recurrence rate in HCC patients with high-risk recurrence factors.

In China, adjuvant TACE is recommended to reduce recurrence rates (2), and its efficacy has been validated by phase III clinical trials (6–8). In addition, other adjuvant therapies, including hepatic arterial infusion chemotherapy (HAIC) and radiotherapy (RT), have also been implemented in clinical practice and are supported by high-quality randomized controlled trials (9–11).

With the advent of the era of immunotherapy, immune checkpoint inhibitors have shown surprising efficacy in this patient population. With the advent of the era of immunotherapy, immune checkpoint inhibitors have demonstrated surprising efficacy in this patient population (12, 13) and have significantly prolonged the recurrence-free survival of these patients in randomized controlled trials (14, 15).

With the emergence of various therapies, there is a need for a deeper understanding of their efficacy. In the absence of head-to-head clinical trials, we conducted a network meta-analysis to indirectly compare the efficacy of endovascular therapy, RT, and immune checkpoint inhibitors.

## 2 Methods

### 2.1 Protocol and registration

This systematic review and network meta-analysis complied with the Preferred Reporting Items for Systematic Review and Meta-Analyses (PRISMA) statement (**Supplementary Table S1**) (16). The protocol for this meta-analysis was registered with PROSPERO (ID: CRD42024576316).

### 2.2 Data sources and search strategies

We conducted a comprehensive search of articles and references in PubMed, Embase, Web of Science, Cochrane Library and Scoups databases. Literature searches were conducted from the time the database was created until October 3, 2024 with the language restricted to English. The detailed search strategies are provided in **Supplementary Table S2**. The studies were independently selected by two researchers who screened the titles and abstracts to identify relevant articles and read the full texts for inclusion. Any disagreements regarding eligibility for inclusion in the analysis were resolved through discussion with the team of researchers.

### 2.3 Eligibility criteria

Published studies that met the following criteria were included:

1. Patients with hepatocellular carcinoma (HCC) who have undergone radical surgery and are assessed preoperatively/intraoperatively as having a moderate to high risk of recurrence include those with the following criteria: multiple lesions ≥ 3, a single lesion with a diameter ≥ 5 cm, microvascular invasion or portal/hepatic vein invasion, and poor differentiation (Edmonson-Steiner grade III/IV);
2. The trial reported one or more of the following clinical outcomes: disease-free survival (DFS), defined as the time from the start of the study to the patient’s first tumour progression or death; 1-year DFS (1-DFS), defined as the proportion of patients in the cohort who have not experienced tumor progression or death within one year after the initiation of the study; overall survival (OS), defined as the time from the start of the study to the onset of death; ≥ 3 grade adverse events (≥ 3 AEs), defined as the incidence of level 3 or higher adverse events as defined by the National Cancer Institute Common Terminology Criteria for Adverse events (CTCAE).
3. The interventions of interest include endovascular therapies such as TACE and HAIC, RT, targeted therapies, and immunotherapy;
4. A randomised controlled trial.

In addition, abstracts, reviews, conference papers, case reports, and animal or *in vitro* studies were excluded.

### 2.4 Data extraction and risk of bias assessment

The research team members prepared a standardised Excel spreadsheet in advance for data extraction. The extracted data included the basic characteristics of the studies, which encompassed the first author, year of publication, country, duration of the study, study phase, clinical trial registration number, inclusion criteria, and sample size; the basic characteristics of the patient cohort, including median age, sex ratio, HBV infection rate, proportion of multiple tumours, proportion of vascular invasion, Child-Pugh liver function classification, and interventions; as well as the outcome data of the studies, including DFS, 1-DFS, OS, and ≥3 AEs.

Two researchers (JiahaoL and YL) independently used the Cochrane tool (RoB2) to assess the methodological quality of included studies. Five domains of the original study were assessed: bias during randomisation, bias in deviation from established interventions, bias in missing outcome data, bias in outcome measurement, and bias in selective reporting. Any disagreements were resolved by consensus through discussions among the team researchers.

### 2.5 Statistical analysis

The primary outcomes were DFS and 1-DFS. The secondary outcomes were OS and ≥3 AEs. We assessed the efficacy of the different treatment regimens by combining the odds ratios (ORs) of the 1-DFS outcomes and the hazard ratios (HRs) of the two survival outcomes with their 95% confidence intervals (95% CIs) and chose whether to use either a random-effects model or a fixed-effects model based on the I^2^ value.

We constructed network plots of various treatment regimens to visually display the direct and indirect comparisons among different treatment options. The gemtc package in R version 4.4.1 was used to fit a consistency model, and we assessed the convergence of the Markov chains using the Gelman-Rubin diagnostic and kernel density estimation plots. In addition, we performed heterogeneity tests. Subsequently, subgroup analysis was conducted for the primary outcome measure, DFS, and meta-regression analysis was performed to explore possible factors influencing the analysis results. All tests were two-sided, and a p-value of <0.05 was considered statistically significant.

## 3 Results

### 3.1 Research screening and characterisation

**Figure 1:**
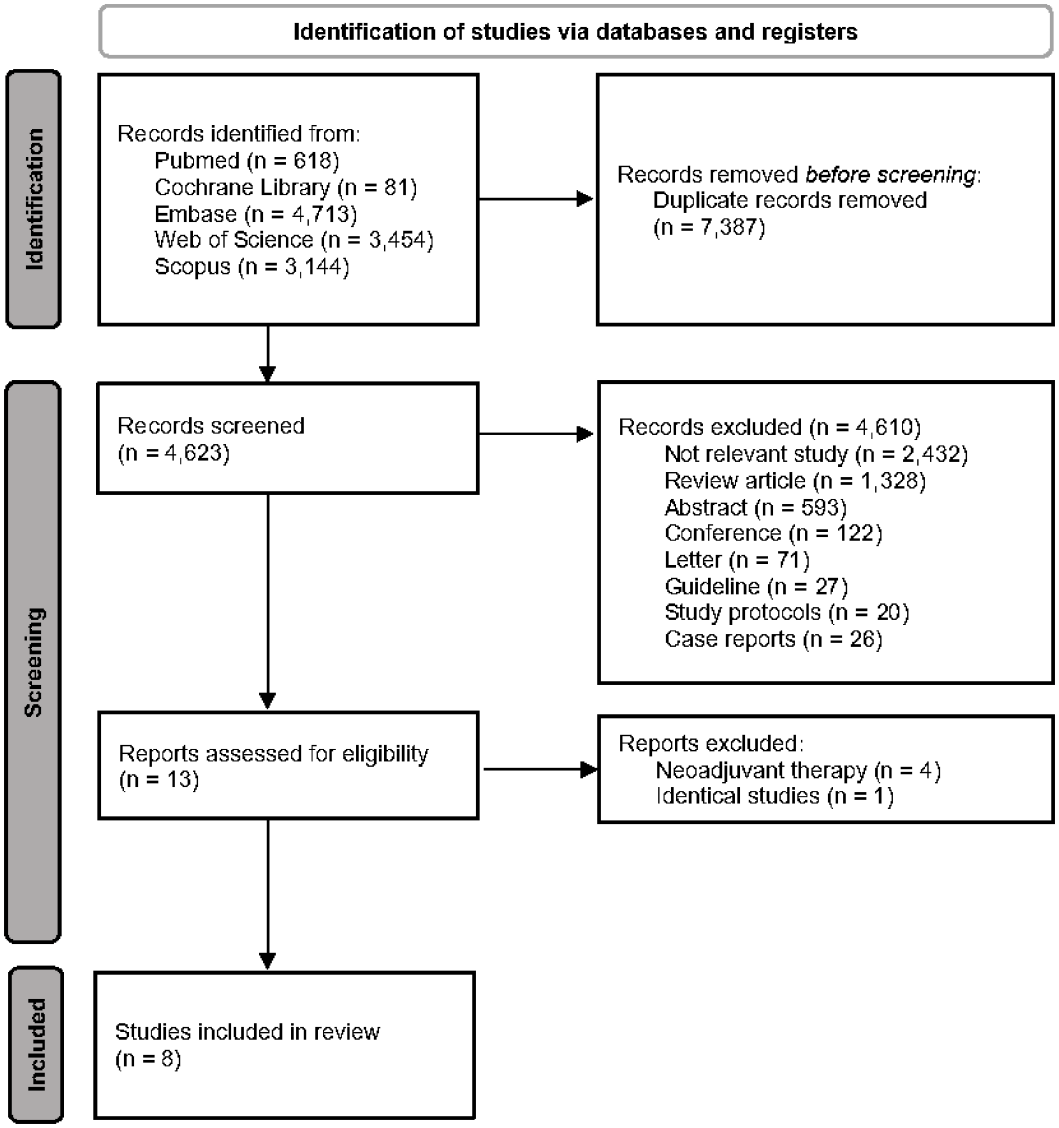
Flow diagram of article search and study selection.

**Figure 2:**
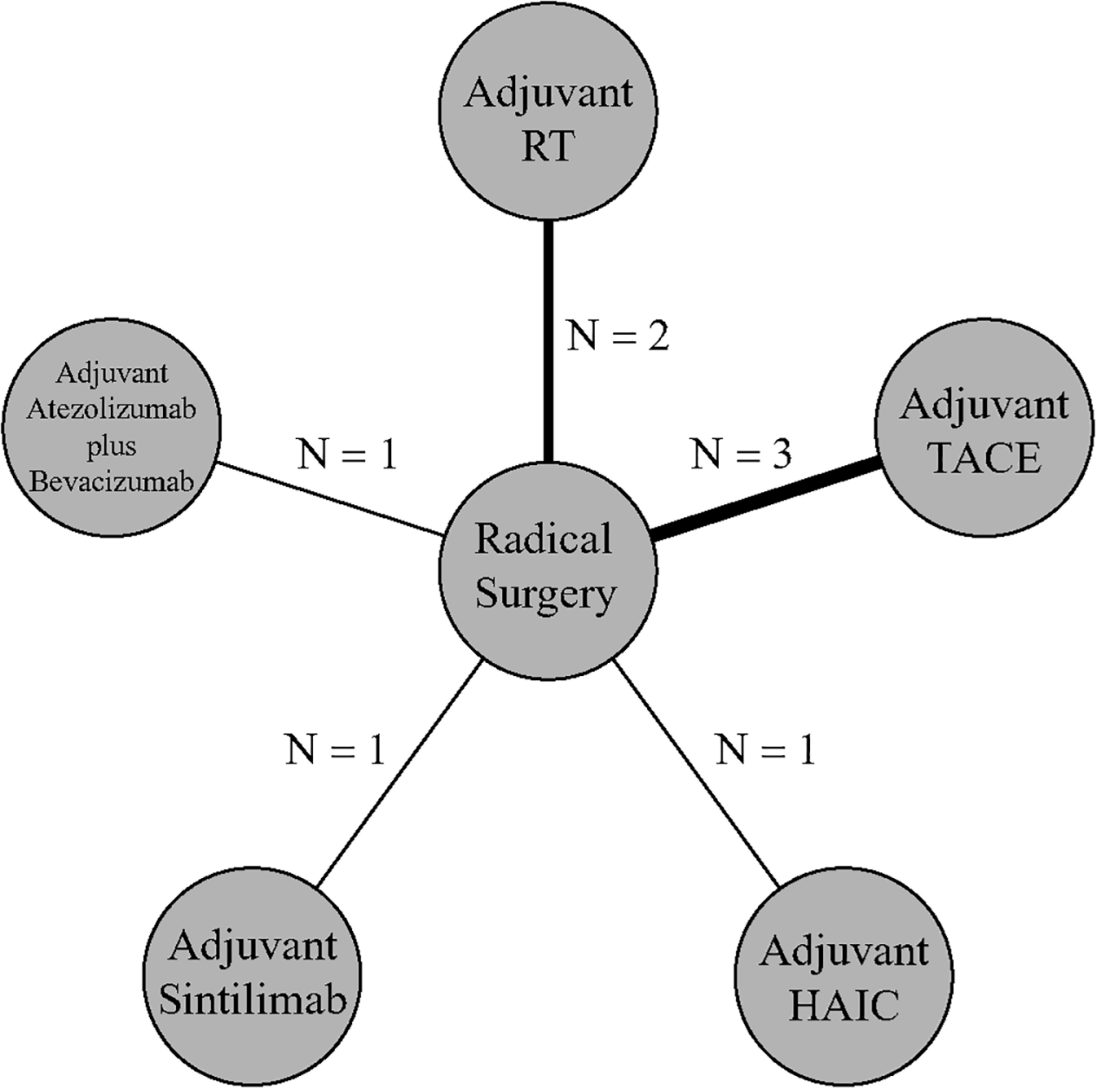
Network diagram.

A total of 12010 articles were retrieved from PubMed, Cochrane Library, Embase, Web of Science and Scoups databases. After removing duplicate records and excluding reviews, abstracts, editorials, conference articles, and studies not relevant to the research content through reading titles and abstracts, a total of 13 articles were subjected to full-text review. Ultimately, 8 studies were included in this meta-analysis (**Figure 1**). Among these studies, 7 were conducted in China, while 1 was conducted across multiple countries. A total of 5 adjunctive treatment regimens were included: TACE (transcatheter arterial chemoembolization), HAIC, RT, atezolizumab + bevacizumab, and sintilimab. In total, 1,909 subjects were enrolled in these studies (**Figure 2**). The basic characteristics of the included studies are presented in **Supplementary Table 3**.

### 3.2 Risk of bias

All studies achieved complete outcome reporting with random allocation. They were all open-label studies. Except for the study by Qin et al., which had some risk in terms of randomization, the other 7 studies exhibited a low risk of bias across the 5 assessed domains (**Supplementary Figure 1**).

### 3.3 DFS

**Figure 3:**
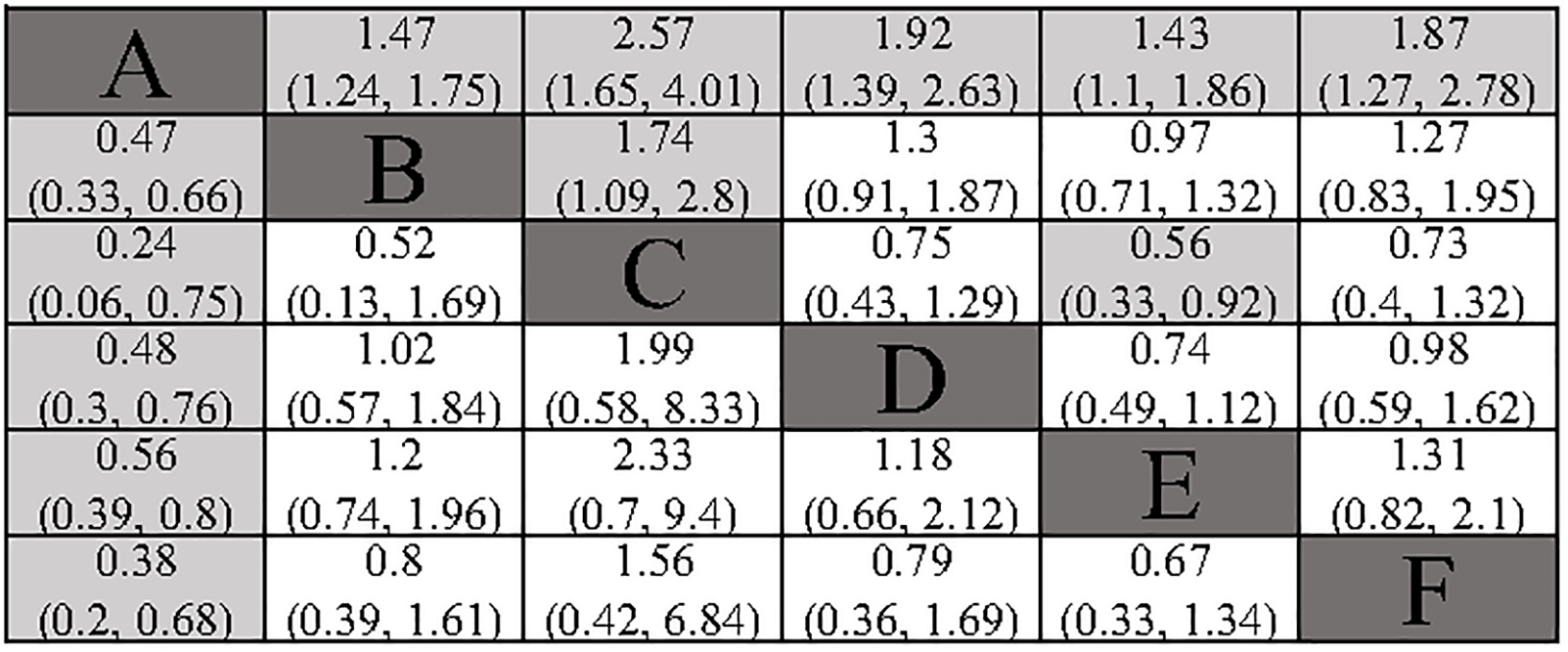
Summary of Network Meta-Analysis. pooled risk ratio (95% confidence interval) for DFS (upper triangle); pooled risk ratio (95% confidence interval) for 1-DFS (lower triangle). A, Radical surgery alone; B, Radical surgery + adjuvant TACE; C, Radical surgery + adjuvant RT; D, Radical surgery + adjuvant HAIC; E, Radical surgery + adjuvant atezolizumab plus bevacizumab; F, Radical surgery + adjuvant sintilimab.

All included studies provided analyzable data on DFS. Compared to surgery alone, the postoperative adjuvant treatment regimens included demonstrated a significant benefit in DFS. In indirect comparisons, RT showed a superior effect in delaying recurrence compared to TACE (HR: 1.74; 95% CI: 1.09, 2.8) and atezolizumab combined with bevacizumab (HR: 0.56; 95% CI: 0.33, 0.92). Meanwhile, no significant statistical differences were observed between TACE, HAIC, atezolizumab combined with bevacizumab, and sintilimab (**Figure 3**). The Gelman-Rubin plot and kernel density estimation indicate a satisfactory convergence of the MCMC chains and demonstrate the robustness of the model (**Supplementary Figure 2**).

All included studies provided analysable data on 1-DFS. All the adjuvant treatment regimens assessed demonstrated a significant benefit in terms of 1-DFS. In the indirect comparison, none of the included adjuvant treatment regimens showed a significant statistical difference (**Figure 3**). The Gelman-Rubin plot and kernel density estimation illustrate that the convergence of the MCMC chains is satisfactory and demonstrate the robustness of the model (**Supplementary Figure 3**).

### 3.4 OS

**Figure 4:**
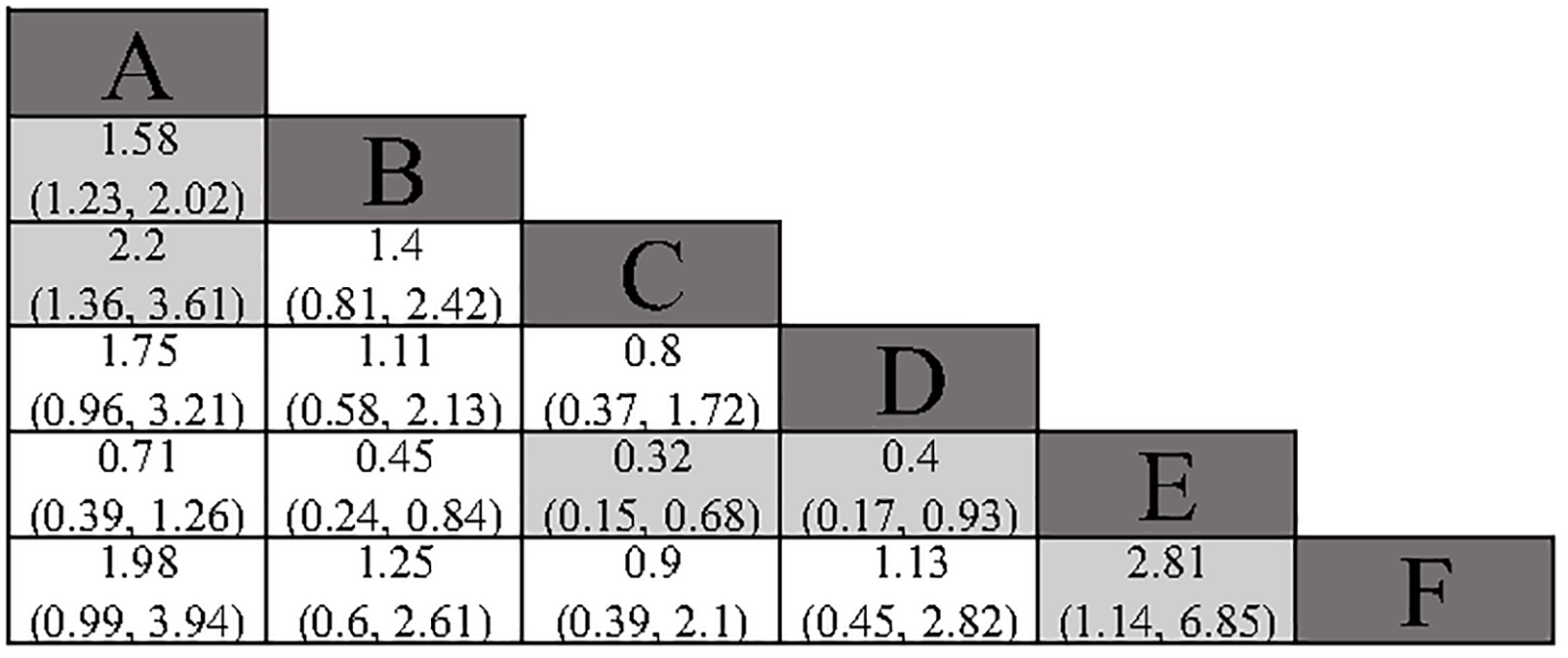
Summary of Network Meta-Analysis. pooled risk ratio (95% confidence interval) for OS. A, Radical surgery alone; B, Radical surgery + adjuvant TACE; C, Radical surgery + adjuvant RT; D, Radical surgery + adjuvant HAIC; E, Radical surgery + adjuvant atezolizumab plus bevacizumab; F, Radical surgery + adjuvant sintilimab.

All included studies reported overall survival (OS) data. Compared to surgery alone, adjuvant TACE (HR: 0.63; 95% CI: 0.50, 0.81) and adjuvant RT (HR: 0.45; 95% CI: 0.28, 0.74) demonstrated a significant OS benefit. Although adjuvant HAIC (HR: 0.57; 95% CI: 0.31, 1.04) and adjuvant sintilimab (HR: 0.51; 95% CI: 0.25, 1.01) did not reach statistical significance, they still showed a favorable trend. In the indirect comparison, RT, HAIC, and sintilimab demonstrated a superior OS benefit compared to atezolizumab plus bevacizumab (**Figure 4**). However, it is important to note that in the studies involving HAIC, atezolizumab plus bevacizumab, and sintilimab, the different study cohorts did not reach the OS endpoint, thereby reducing the credibility of these results. The Gelman-Rubin plot and kernel density estimation illustrate that the convergence of the MCMC chains is satisfactory and demonstrate the robustness of the model (**Supplementary Figure 4**).

### 3.5 AEs

The assessment of AEs is descriptive in nature. In the studies involving TACE, RT, and HAIC, most of the complications related to adjuvant therapy were classified as grade 1-2, indicating that the patients were generally tolerable. None of the six studies reported AEs that would change the treatment outcomes. In the IMbrave050 trial, 41% of patients in the postoperative group receiving a combination of atezolizumab and bevacizumab experienced grade 3 or 4 AEs, compared to an incidence of just 13% in the postoperative active monitoring group. Notably, there was a significant increase in the rates of hypertension, proteinuria, and decreased platelet count among those in the combination therapy group. In patients receiving postoperative treatment with atezolizumab in combination with bevacizumab, 63% experienced immune-mediated AEs of any grade, with grade 3 or 4 AEs reaching 10%. The most common events among these were hepatitis and hypothyroidism. It is noteworthy that the combination of atezolizumab and bevacizumab may increase the incidence of bleeding events. Furthermore, any grade of AEs leading to the discontinuation of atezolizumab and bevacizumab occurred in 9% of patients. In the study by Wang et al., 63.9% of patients in the sintilimab group experienced treatment-related AEs of any grade, with 12.4% experiencing grade 3 or 4 treatment-related AEs. Similar to the IMbrave050 trial, 8.1% of patients discontinued treatment due to AEs, with elevated ALT levels being a major contributing factor. **Table 1** summarizes the incidence of select ≥3 AEs.

**Table 1:** Descriptive assessment of ≥3 AEs.

Study	Treatment	Fever	Fatigue	Pain	Nausea/vomiting	Gastric ulcer	Increase in ALT/AST	Increase in bilirubin	Anemia	Leukopenia	Decreased platelet vount	Hypertension	Proteinuria	Pruritus
Wang 2018	TACE	NA	NA	0%	0%	NA	0%	0%	NA	0%	NA	NA	NA	NA
Wei 2018	TACE	3%	2%	2%	1%	NA	NA	NA	2%	5%	3%	NA	NA	NA
Sun 2019	RT	NA	15%	NA	13%	13%	12%	13%	NA	NA	NA	NA	NA	NA
Shi 2022	RT	0%	0%	0%	0%	NA	0%	0%	0%	0%	0%	NA	0%	NA
Li 2023	HAIC	0%	NA	0%	0%	NA	0%	0%	0%	0%	NA	NA	NA	NA
Qin 2023	Atezolizumab plus bevacizumab	0%	NA	NA	NA	NA	<1%	<1%	NA	NA	5%	18%	9%	<1%
Wang 2024	Sintilimab	1%	3%	NA	NA	NA	9%	3%	6%	0%	3%	NA	0%	2%

### 3.6 Probability of ranking

**Figure 5:**
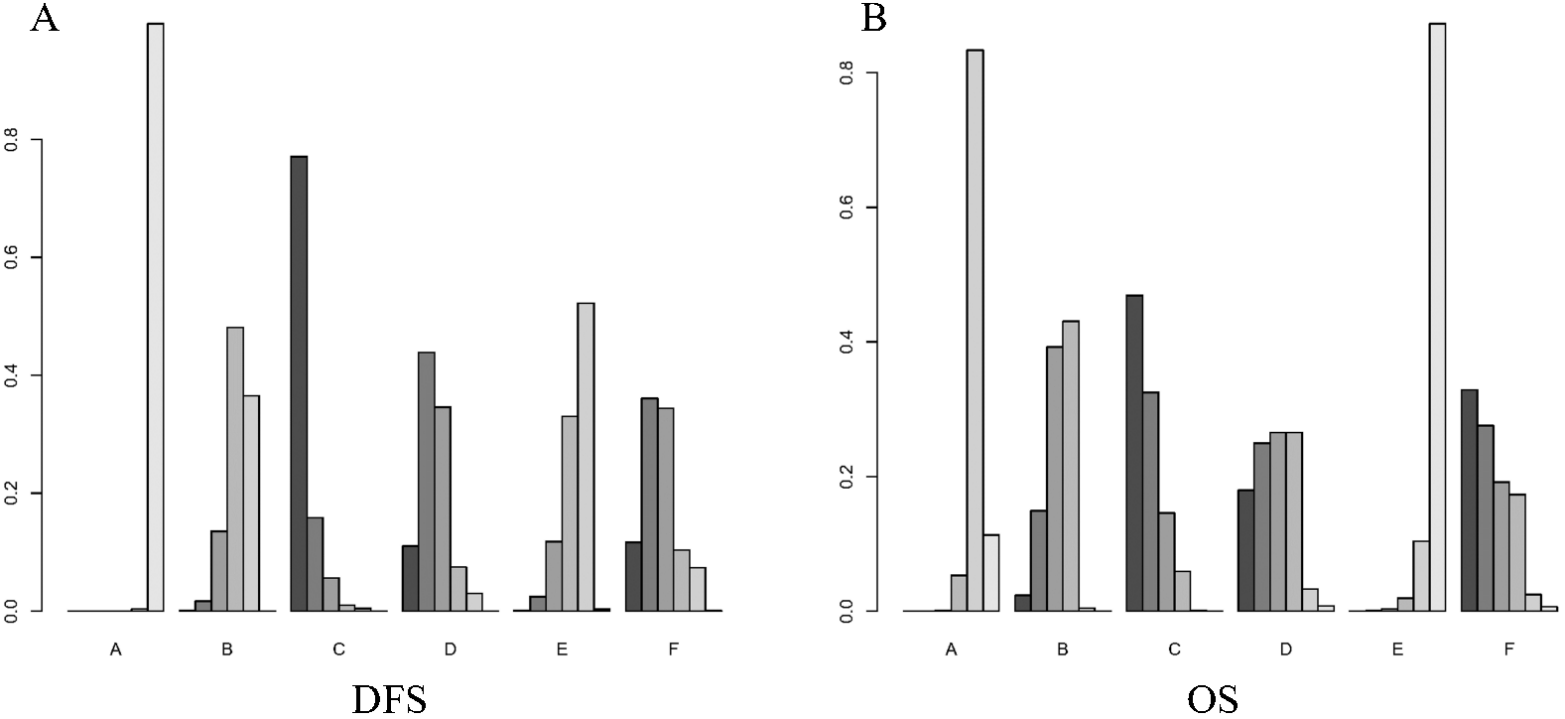
Bayesian ranking plot of treatment. **(A)** DFS; **(B)** OS. A, Radical surgery alone; B, Radical surgery + adjuvant TACE; C, Radical surgery + adjuvant RT; D, Radical surgery + adjuvant HAIC; E, Radical surgery + adjuvant atezolizumab plus bevacizumab; F, Radical surgery + adjuvant sintilimab.

**Figure 5** shows the Bayesian ranking of the different outcomes for the different treatment options. **Supplementary Table S4** summarises the ranking results. The Bayesian ranking results were consistent with the results of the HR analyses. Furthermore, we conducted a comprehensive assessment of the included treatment regimens based on the SUCRA values, as presented in **Table 2**.

**Table 2:** Ranking based on SUCRA values.

Ranks	DFS			OS
	Treatment	SUCRA(%)	Treatment	SUCRA(%)
Best	C	93.63	C	84.01
2^nd^	D	70.48	F	73.82
3^rd^	F	66.82	D	65.09
4^th^	B	36.13	B	55.13
5^th^	E	32.86	A	18.82
Worst	A	0.08	E	3.13

A, Radical surgery alone; B, Radical surgery + adjuvant TACE; C, Radical surgery + adjuvant RT; D, Radical surgery + adjuvant HAIC; E, Radical surgery + adjuvant atezolizumab plus bevacizumab; F, Radical surgery + adjuvant sintilimab.

### 3.7 Heterogeneity assessment

**Figure 6:**
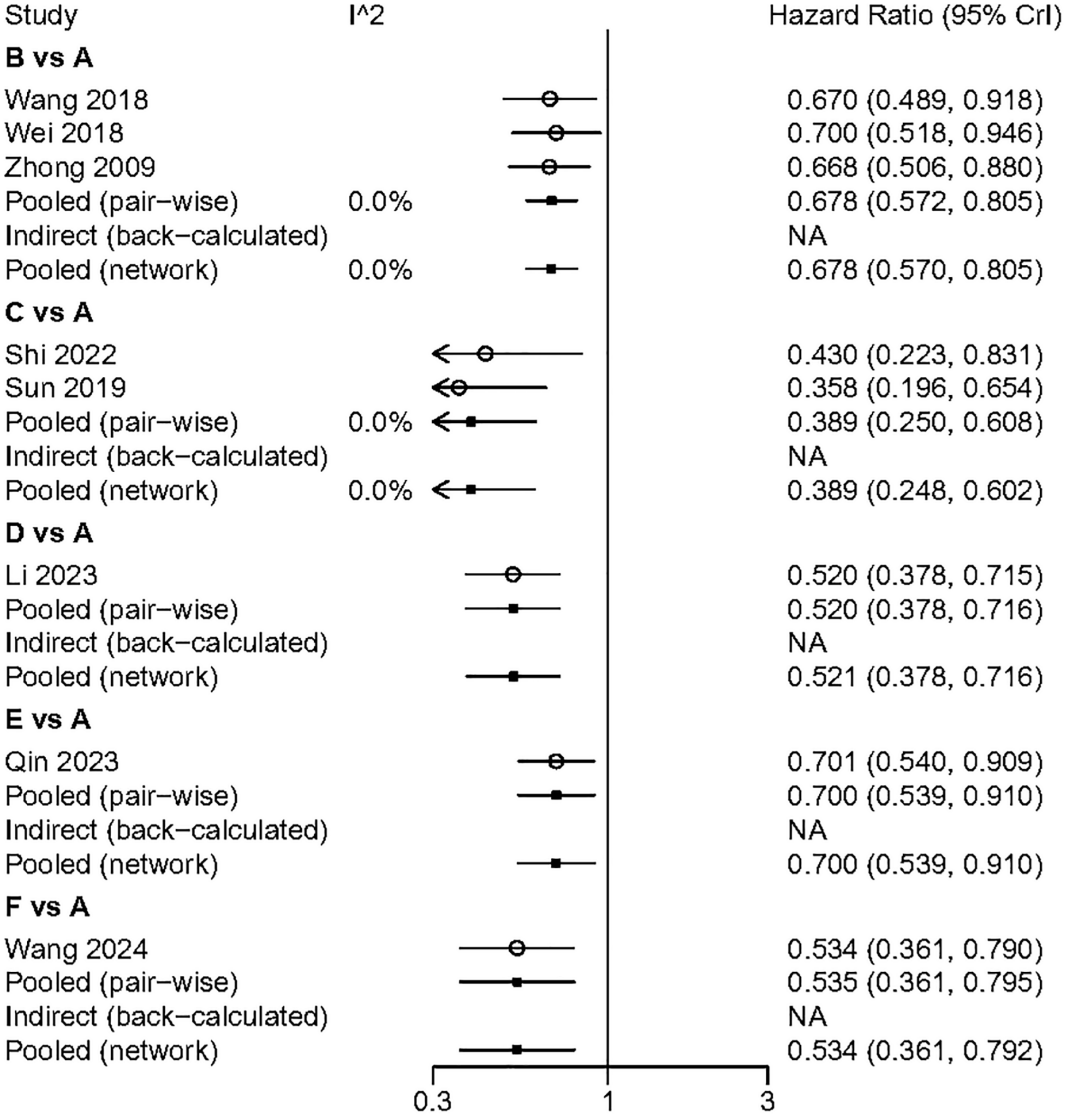
The pairwise meta-analysis of DFS. A. Radical surgery alone; B, Radical surgery + adjuvant TACE; C, Radical surgery + adjuvant RT; D, Radical surgery + adjuvant HAIC; E, Radical surgery + adjuvant atezolizumab plus bevacizumab; F, Radical surgery + adjuvant sintilimab.

**Figure 7:**
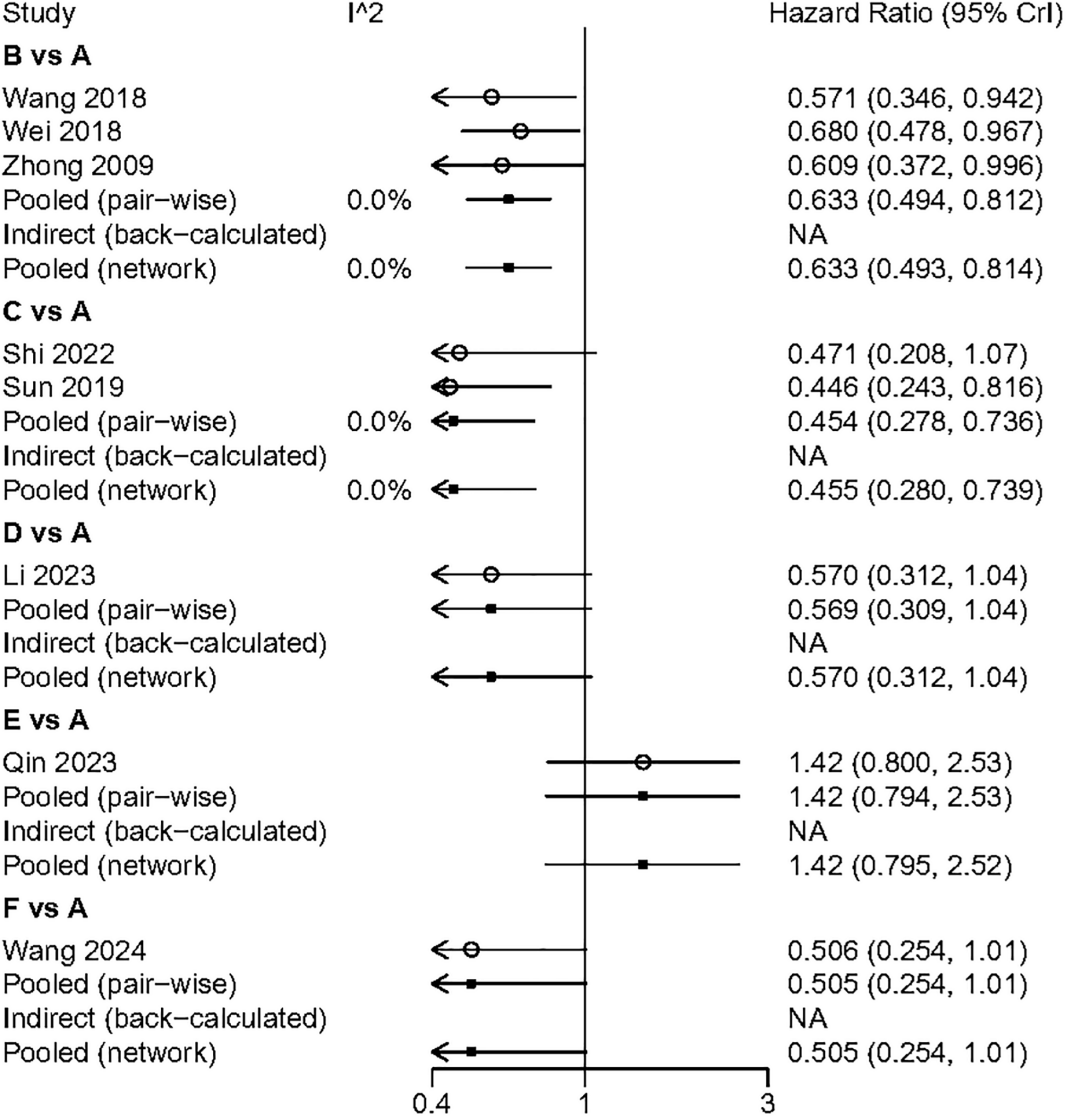
The pairwise meta-analysis of OS. A, Radical surgery alone; B, Radical surgery + adjuvant TACE; C, Radical surgery + adjuvant RT; D, Radical surgery + adjuvant HAIC; E, Radical surgery + adjuvant atezolizumab plus bevacizumab; F, Radical surgery + adjuvant sintilimab.

We performed a pairwise meta-analysis of the outcomes from different treatment regimens. The results indicated that there was no significant heterogeneity in the combined DFS and OS (I² = 0%) (**Figures 6**, **7**).

### 3.8 Subgroup analyses

**Figure 8:**
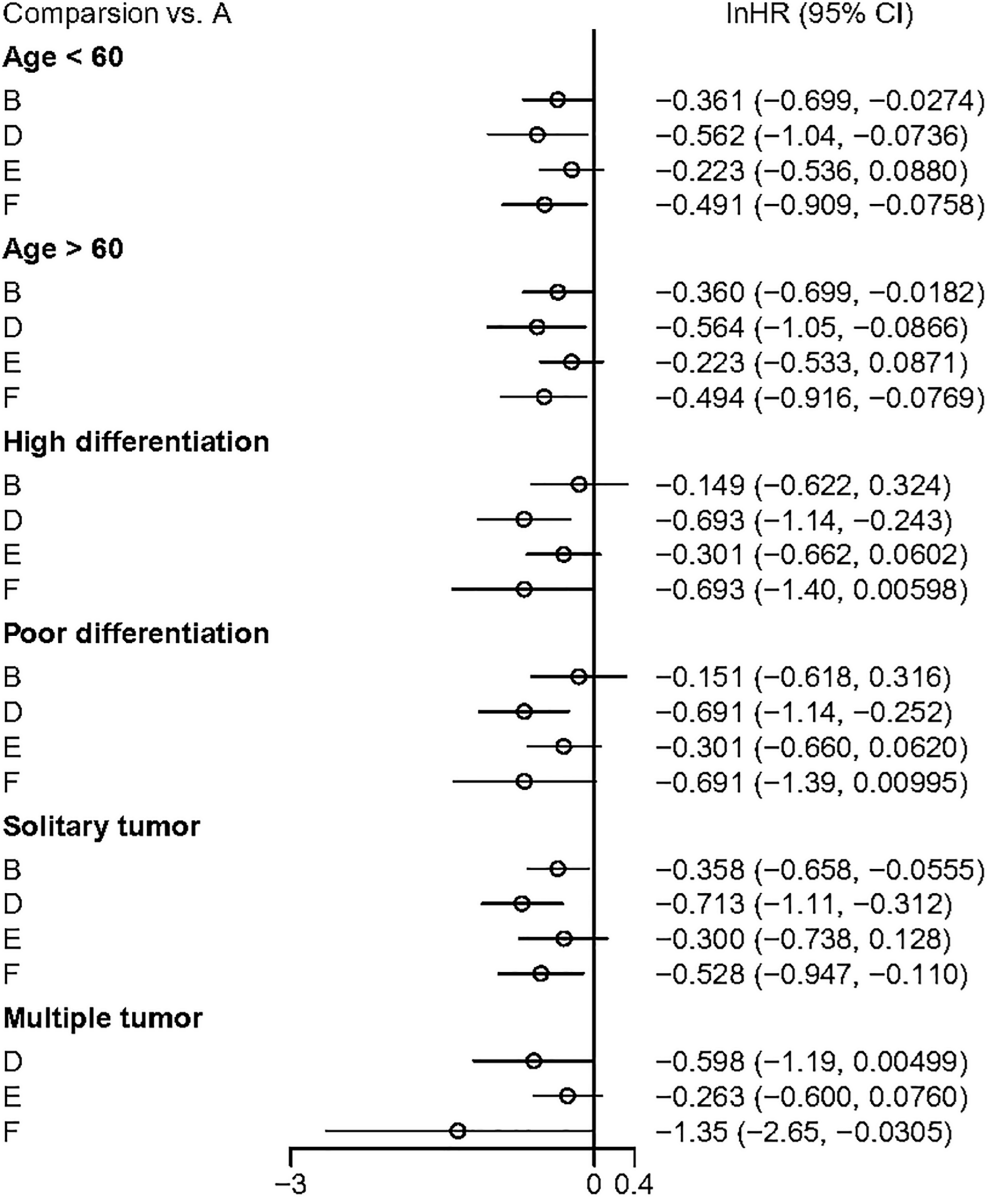
The outcome of subguoup analyses. A, Radical surgery alone; B, Radical surgery + adjuvant TACE; C, Radical surgery + adjuvant RT; D, Radical surgery + adjuvant HAIC; E, Radical surgery + adjuvant atezolizumab plus bevacizumab; F, Radical surgery + adjuvant sintilimab.

Subgroup analyses of DFS results were conducted based on the age of the study cohorts, tumor differentiation, and tumor quantity (**Figure 8**). The results showed that both adjuvant HAIC and sintilimab provided significant DFS benefits in these patients.

### 3.9 Network regression analysis

Network meta-regression analysis of disease-free survival (DFS) results revealed that variables such as study duration, sample size, patient age, gender, HBV infection, multiple tumours, and vascular invasion were not significantly associated with the combined DFS outcomes (**Table 3**).

**Table 3:** Meta-regression results.

Covariate	Coefficient (95% CI)
Starting year	0.037 (-0.331, 0.405)
Sample size	0.050 (-0.770, 0.888)
Age of patients	-0.003 (-0.382, 0.375)
Sex of patients	-0.002 (-0.322, 0.320)
HBV infection	-0.047 (-0.651, 0.556)
Number of tumors	-0.037 (-0.383, 0.309)
Vascular invasion	0.042 (-0.303, 0.388)

## 4 Discussion

In recent years, there has been rapid advancement in the treatment of hepatocellular carcinoma HCC. Currently, localized regional therapies and combination treatment strategies based on these local therapies are important options for patients. Endovascular therapies such as TACE and HAIC are widely used and supported by high-quality randomized controlled trials RCTs (17, 18). Combination therapies based on TACE are being extensively explored in various clinical scenarios (19). HAIC is another endovascular therapy that exerts tumor control by continuously infusing chemotherapy drugs directly into the tumor lesion. In clinical practice, HAIC is often employed to treat HCC patients with a high tumor burden and those in more advanced stages of the disease. Based on the FOLFOX chemotherapy regimen, HAIC has demonstrated significant clinical efficacy in certain patient subgroups, including those with large tumor burdens, advanced HCC, and portal vein invasion (18, 20, 21). The application of immune checkpoint inhibitors has ushered in a new era of immunotherapy for HCC (22–24). Although some guidelines recommend the combination of atezolizumab and bevacizumab as the preferred treatment for advanced HCC (25), the relatively high incidence of adverse reactions associated with this regimen remains a cause for concern (26). So far, radical tumour resection remains the primary curative treatment for patients with early-stage HCC. However, the high postoperative recurrence rate continues to be a significant factor affecting patient prognosis, particularly among those with high-risk recurrence factors. Such patients often exhibit more aggressive tumour phenotypes and biological behaviour (27, 28). Therefore, it is crucial to implement necessary measures to reduce the recurrence rate (20).

This network meta-analysis compared the effects of endovascular therapy, radiotherapy, and immunotherapy in patients with HCC who underwent radical surgery and presented with high-risk recurrence factors. The main findings are summarised as follows: in patients with HCC exhibiting high-risk recurrence factors, adjuvant endovascular therapy, radiotherapy, and immunotherapy all demonstrated significant efficacy in controlling recurrences. In indirect comparisons, adjuvant radiotherapy showed superior recurrence control compared to TACE and the combination of atezolizumab and bevacizumab; however, no statistically significant differences were observed between adjuvant radiotherapy and adjuvant HAIC or sintilimab. In direct comparisons regarding OS, postoperative TACE and radiotherapy exhibited improved OS outcomes compared to surgery alone. In indirect comparisons of OS, postoperative radiotherapy, HAIC, and sintilimab provided superior OS benefits compared to the combination of atezolizumab and bevacizumab. Moreover, sintilimab and the combination of atezolizumab and bevacizumab were associated with a tendency towards a higher incidence of serious AEs.

### 4.1 Research strengths and limitations

This study conducted a comprehensive analysis of the effectiveness and safety of five adjuvant therapeutic approaches by investigating three outcome indicators: DFS, OS, and AEs. Prior to initiating the research, we established an extensive search strategy, ensuring that the data incorporated were thorough and derived from high-quality randomised controlled trials, which further enhanced the credibility of the study. Additionally, we performed subgroup analyses and meta-regression analyses to provide further recommendations for clinical decision-making for this patient population.

However, this study does have limitations. Firstly, the potential variations in study protocols, patient populations, and interventions among the included studies make it challenging to draw definitive conclusions. For instance, the inclusion of different adjuvant therapies and patient cohorts with varying baseline characteristics may introduce confounding factors that cannot be fully addressed, even though network meta-regression analyses may have partially mitigated this bias. Secondly, among the eight studies included, seven were conducted in China, while only one was a multinational study. This raises concerns about the generalizability of the results to populations outside of China, as regional differences in the etiology, genetics, and healthcare practices related to hepatocellular carcinoma. Thirdly, although all studies reported OS, the studies conducted by Li et al. (11), Qin et al. (15), and Wang et al. (14) did not reach the OS endpoint. This weakens the overall conclusions regarding the survival benefits of these therapies and reduces the credibility of the results, especially concerning the indirect comparisons in the network meta-analysis. Therefore, the combined outcomes may be subject to considerable bias, and the analysis results should be interpreted with caution.

### 4.2 Study implications and prospects

There remains ongoing debate regarding adjuvant therapy for HCC. Guidelines from Western countries and some East Asian nations do not recommend or explicitly endorse adjuvant treatment for patients with high-risk recurrence factors (29–32). Postoperative active surveillance can assist clinicians in identifying early tumor recurrences, which may be treated through subsequent radical resection. However, portal hypertension, insufficient functional reserve of the remaining liver, and technical difficulties may render repeated resections challenging and risky (33). Therefore, discussing suitable and effective adjuvant therapy options is of paramount importance.

In China, adjuvant TACE is recommended for patients to reduce recurrence rates (2). This study supports the utility of radiotherapy, HAIC, and immunotherapy in adjuvant treatment, contributing to updates in the guidelines. The findings demonstrate that external beam radiotherapy shows significant potential for the management of HCC patients with high-risk recurrence factors. With the advancement of new technologies such as three-dimensional conformal radiotherapy, intensity-modulated radiation therapy, and stereotactic body radiation therapy, it is now possible to enhance the radiation dose delivered to target areas while better sparing adjacent healthy liver tissue. This significantly facilitates the clinical application of radiotherapy techniques (9, 34). Furthermore, the results of this study demonstrate that adjuvant immunotherapy is not inferior to local regional therapies in terms of efficacy, thereby promoting the application of immunotherapy in a broader range of clinical scenarios. However, the higher incidence of adverse reactions associated with immune checkpoint inhibitors and their safety profile remain important concerns that warrant attention.

At the same time, the results of this study highlight several issues that need to be addressed in the future. Firstly, current research provides very limited data on long-term outcomes. While DFS is important, a more comprehensive exploration of overall survival over longer follow-up periods would be more beneficial. Secondly, certain adjuvant therapies, particularly the combination of atezolizumab and bevacizumab, exhibit a high incidence of adverse reactions, raising concerns regarding the safety of these treatments. The significantly increased rates of hypertension, proteinuria, and thrombocytopenia, as well as immune-mediated adverse effects such as hepatitis and hypothyroidism in these patient populations, underscore the need for a more careful risk-benefit analysis in clinical practice. Thirdly, future trials should aim to include direct head-to-head comparisons of the most promising treatments identified in this study whenever possible, to reduce the uncertainty associated with indirect comparisons. Finally, for certain therapies lacking reliable Phase III clinical trial data, further research should be conducted to reinforce the conclusions drawn from this study.

## 5 Conclusion

In this systematic review and network meta-analysis, we evaluated the efficacy and safety of various adjuvant treatment regimens for HCC patients with high-risk recurrence factors. The results indicate that adjuvant immunotherapy provides a comparable effect in delaying tumor recurrence when compared to local regional therapies; however, its safety profile remains a significant concern, necessitating the establishment of stringent usage criteria before clinical application. Adjuvant radiotherapy demonstrated superior efficacy in delaying tumor recurrence compared to adjuvant TACE, but further support from Phase III clinical trial evidence is required. Additionally, the long-term effects of HAIC and immunotherapy in this patient population warrant further investigation.

## Data Availability

The original contributions presented in the study are included in the article/**Supplementary Material**. Further inquiries can be directed to the corresponding author.
