# Supplementary material for: Comparison of adjuvant treatment regimens for high-risk hepatocellular carcinoma: a Bayesian network meta analysis and systematic review
Source: Front Immunol. 2024 Nov 11;15:1487353. doi: 10.3389/fimmu.2024.1487353 (PMC11586331; doi:10.3389/fimmu.2024.1487353)
Supplement: Supplementary file 2 [file Presentation1.pptx]

## Slide 1
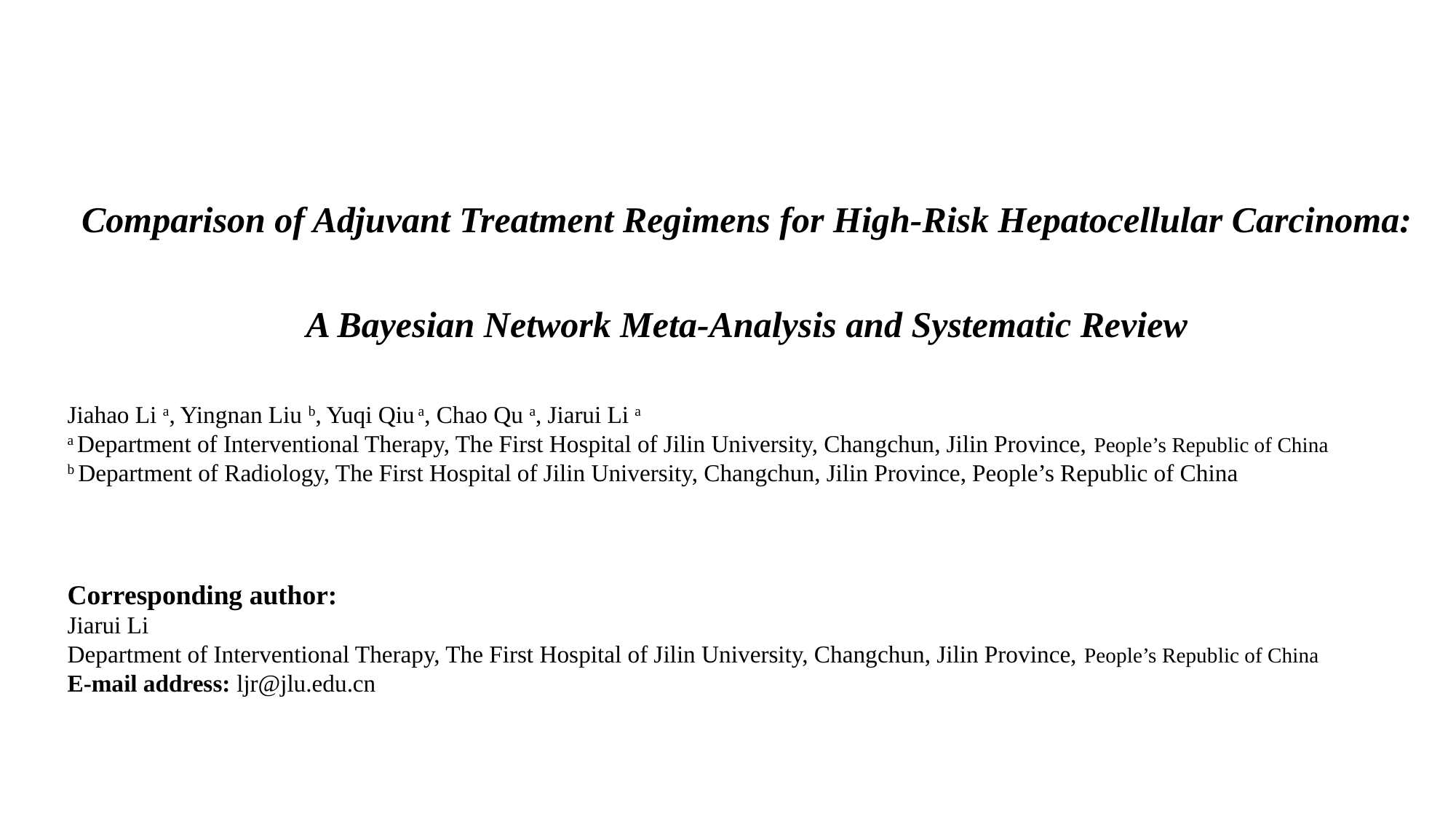

Comparison of Adjuvant Treatment Regimens for High-Risk Hepatocellular Carcinoma: A Bayesian Network Meta-Analysis and Systematic Review
Jiahao Li a, Yingnan Liu b, Yuqi Qiu a, Chao Qu a, Jiarui Li a
a Department of Interventional Therapy, The First Hospital of Jilin University, Changchun, Jilin Province, People’s Republic of China
b Department of Radiology, The First Hospital of Jilin University, Changchun, Jilin Province, People’s Republic of China
Corresponding author:
Jiarui Li
Department of Interventional Therapy, The First Hospital of Jilin University, Changchun, Jilin Province, People’s Republic of China
E-mail address: ljr@jlu.edu.cn

## Slide 2
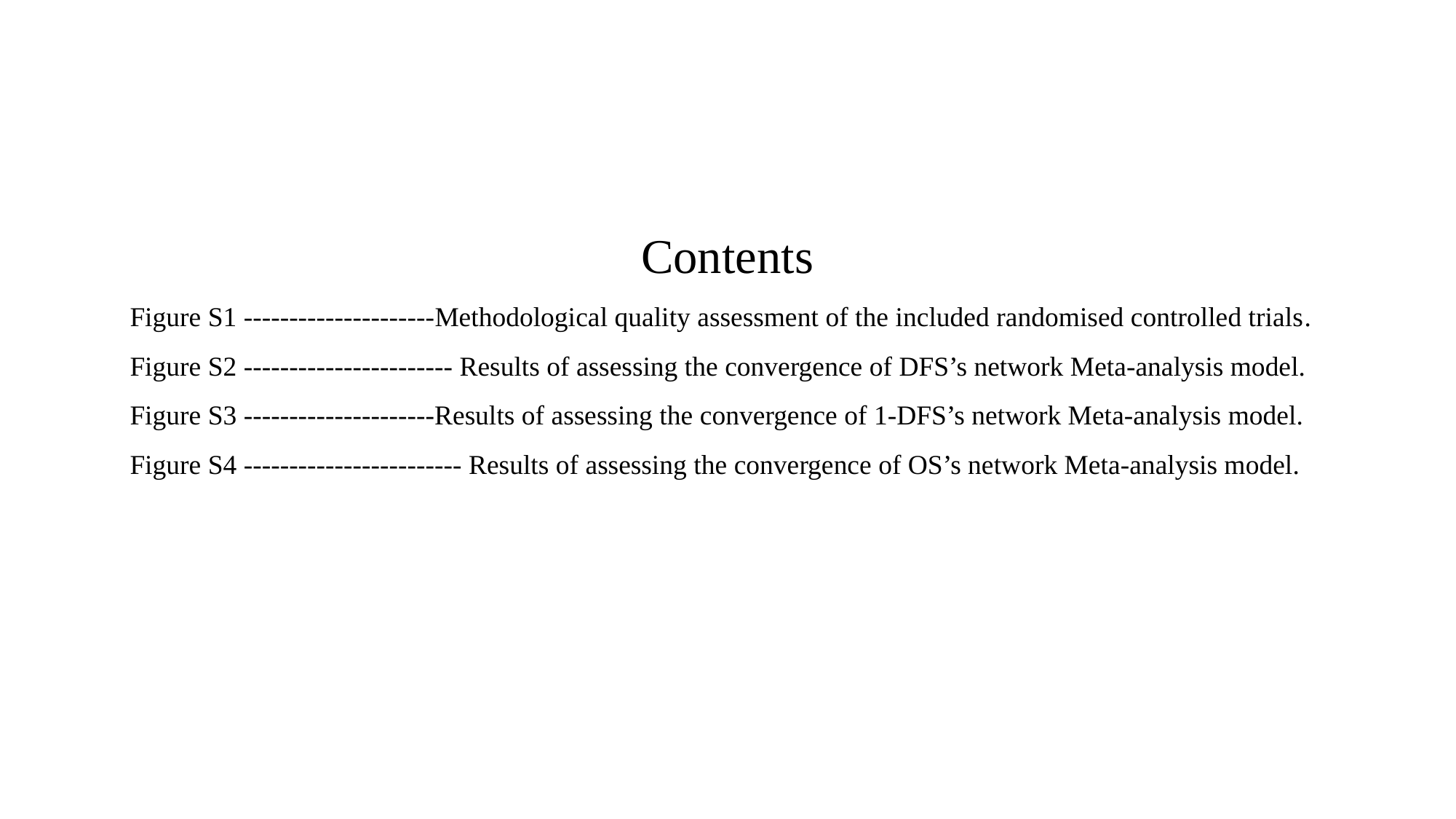

Contents
Figure S1 ---------------------Methodological quality assessment of the included randomised controlled trials.
Figure S2 ----------------------- Results of assessing the convergence of DFS’s network Meta-analysis model.
Figure S3 ---------------------Results of assessing the convergence of 1-DFS’s network Meta-analysis model.
Figure S4 ------------------------ Results of assessing the convergence of OS’s network Meta-analysis model.

## Slide 3
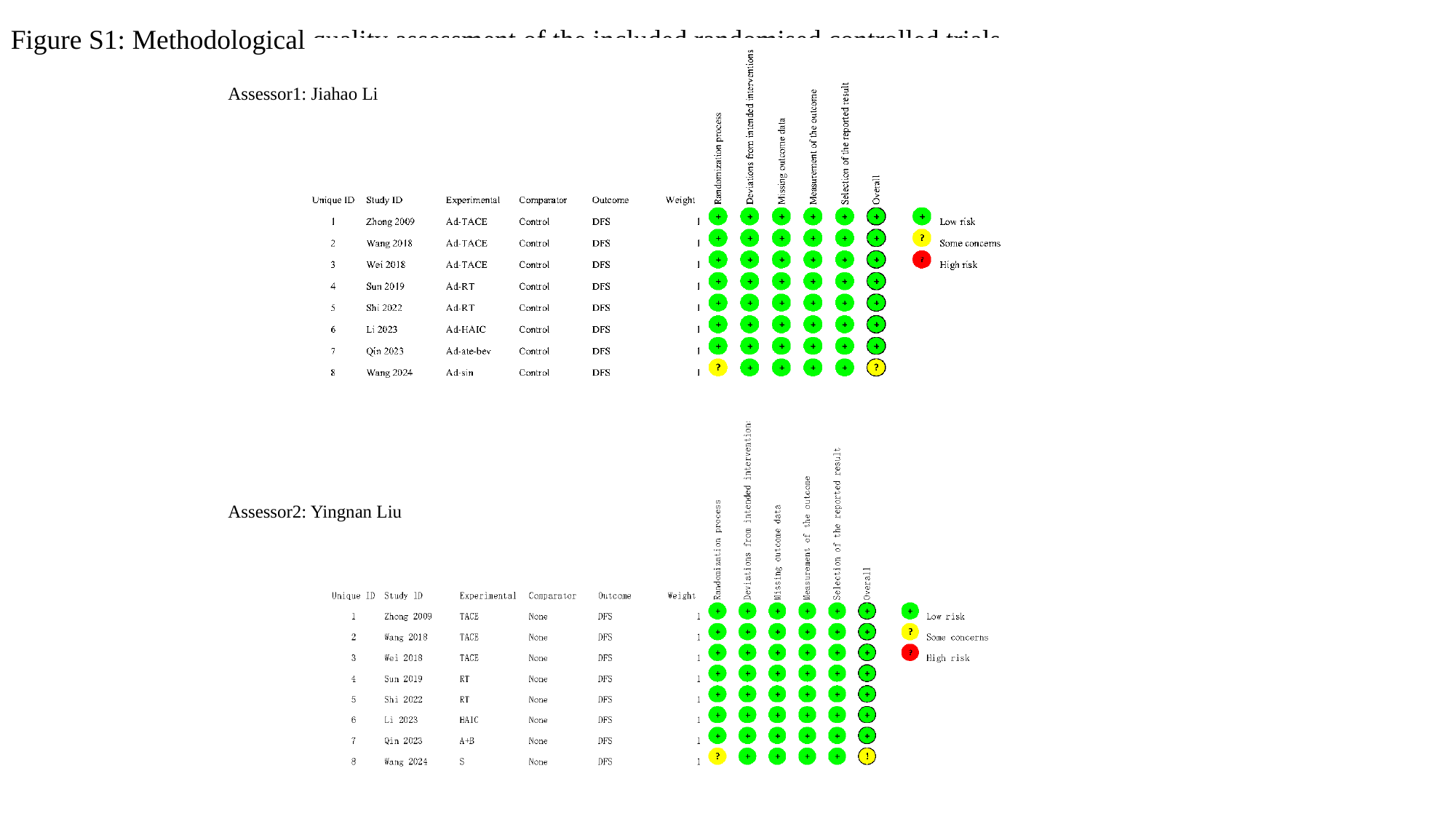

Figure S1: Methodological quality assessment of the included randomised controlled trials.
Assessor1: Jiahao Li
Assessor2: Yingnan Liu

## Slide 4
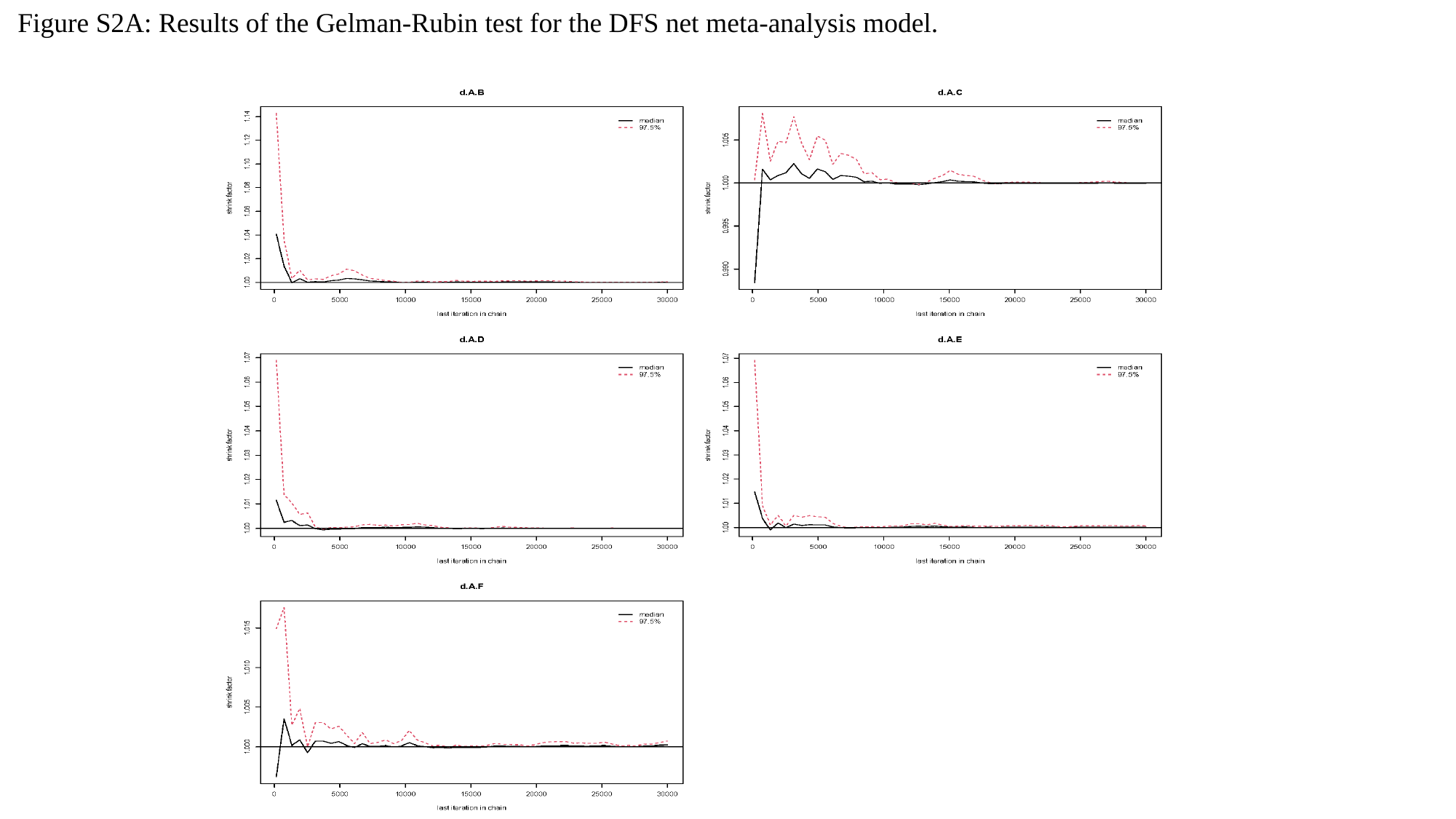

Figure S2A: Results of the Gelman-Rubin test for the DFS net meta-analysis model.

## Slide 5
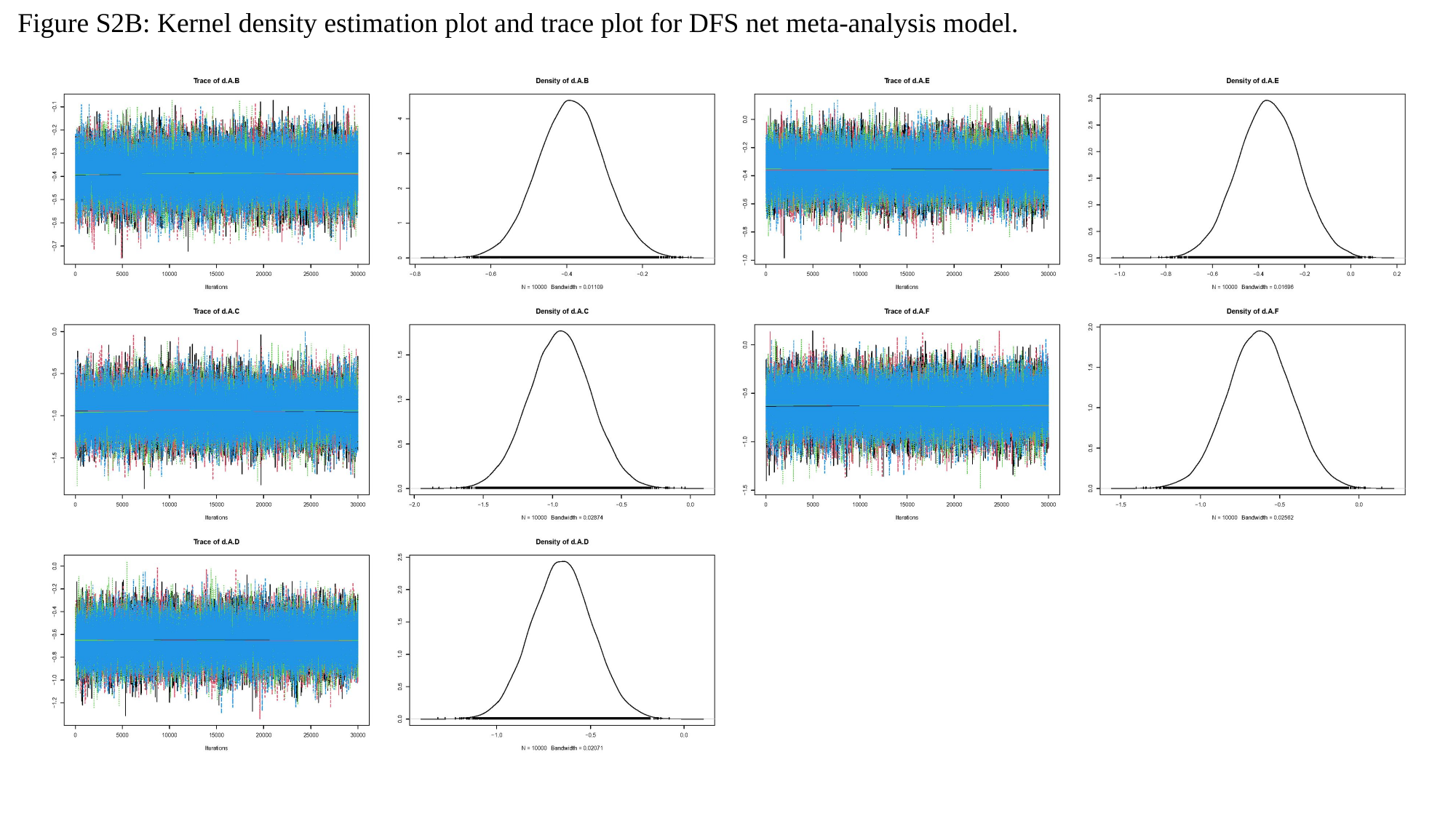

Figure S2B: Kernel density estimation plot and trace plot for DFS net meta-analysis model.

## Slide 6
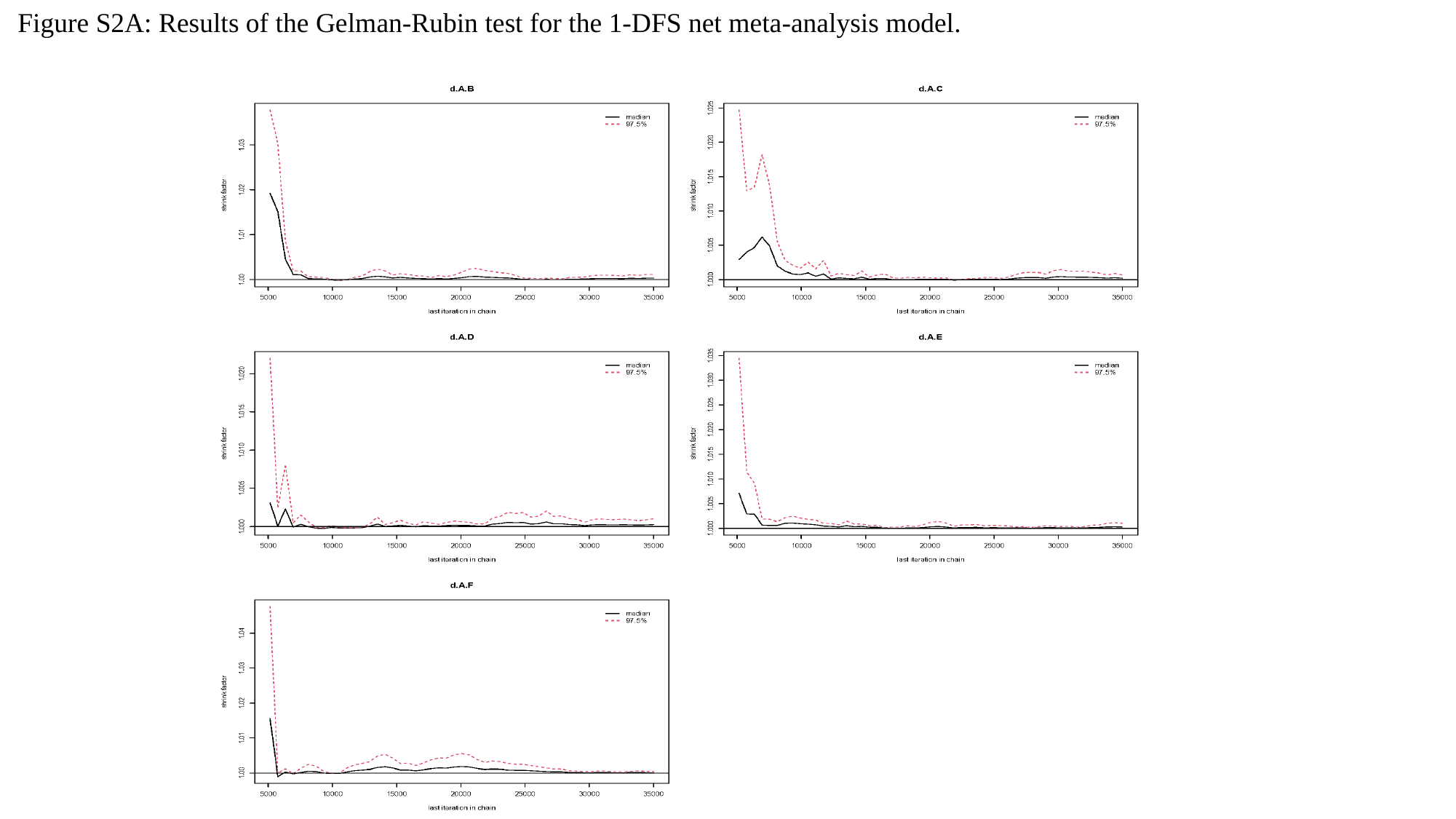

Figure S2A: Results of the Gelman-Rubin test for the 1-DFS net meta-analysis model.

## Slide 7
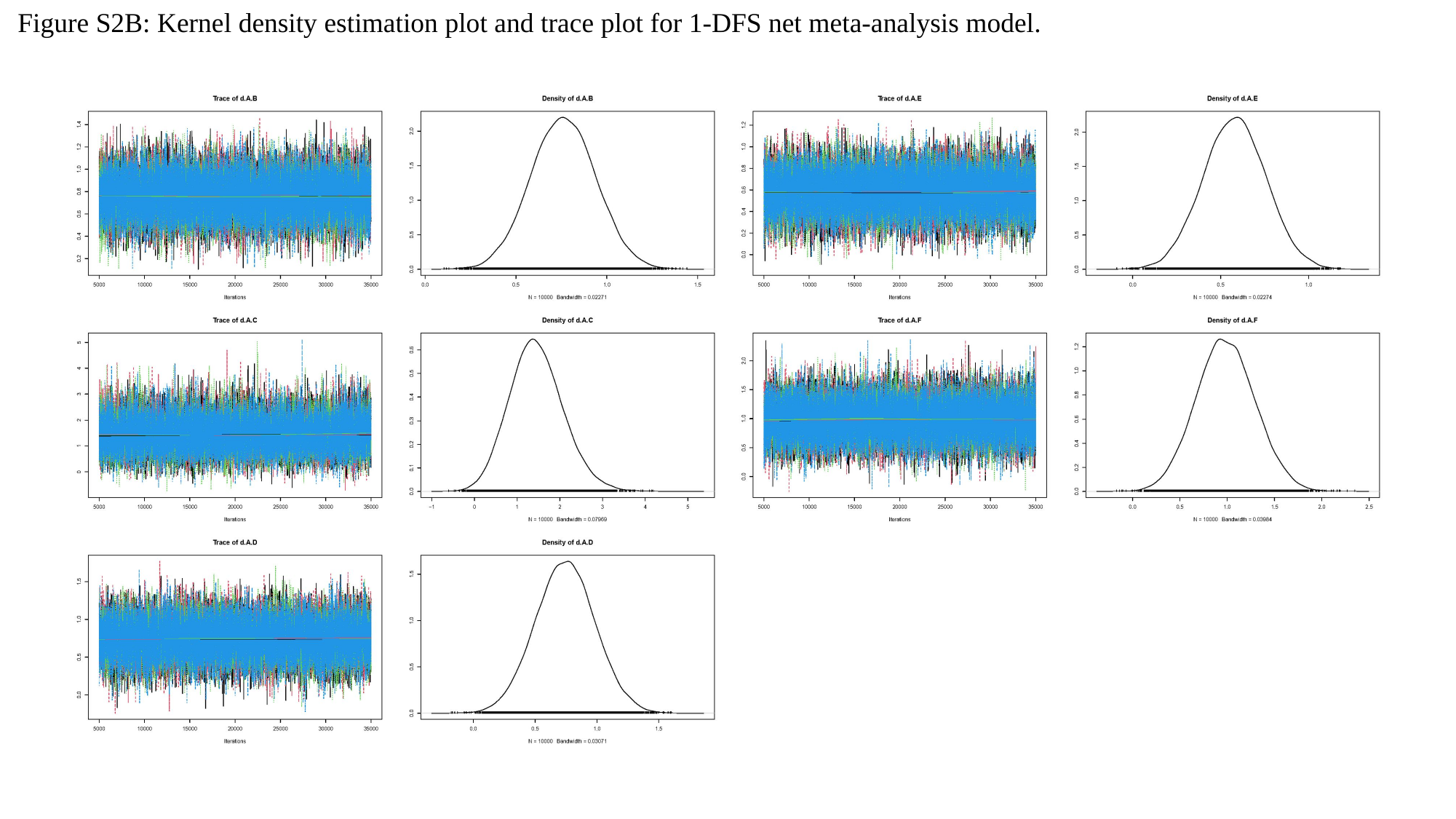

Figure S2B: Kernel density estimation plot and trace plot for 1-DFS net meta-analysis model.

## Slide 8
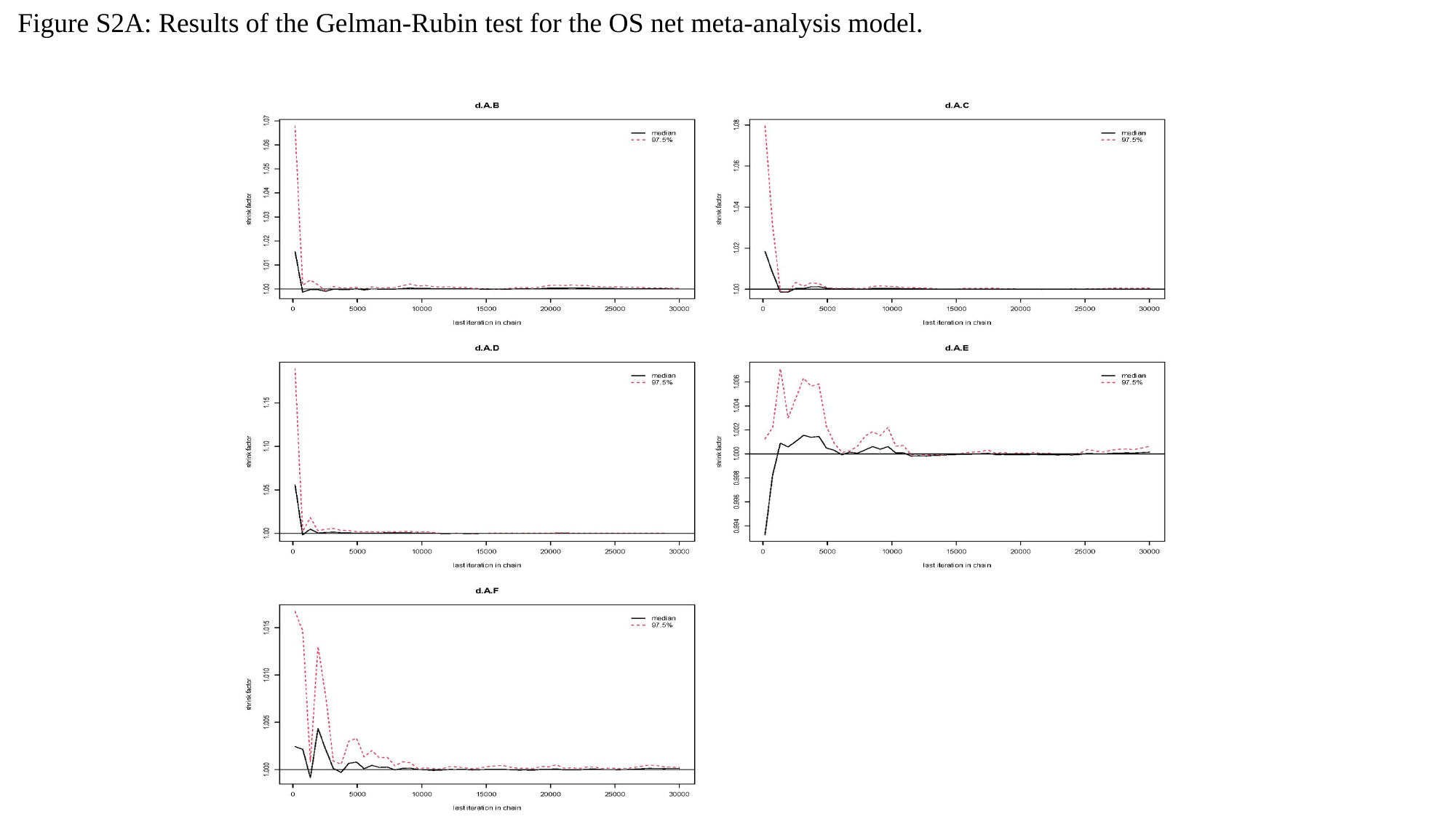

Figure S2A: Results of the Gelman-Rubin test for the OS net meta-analysis model.

## Slide 9
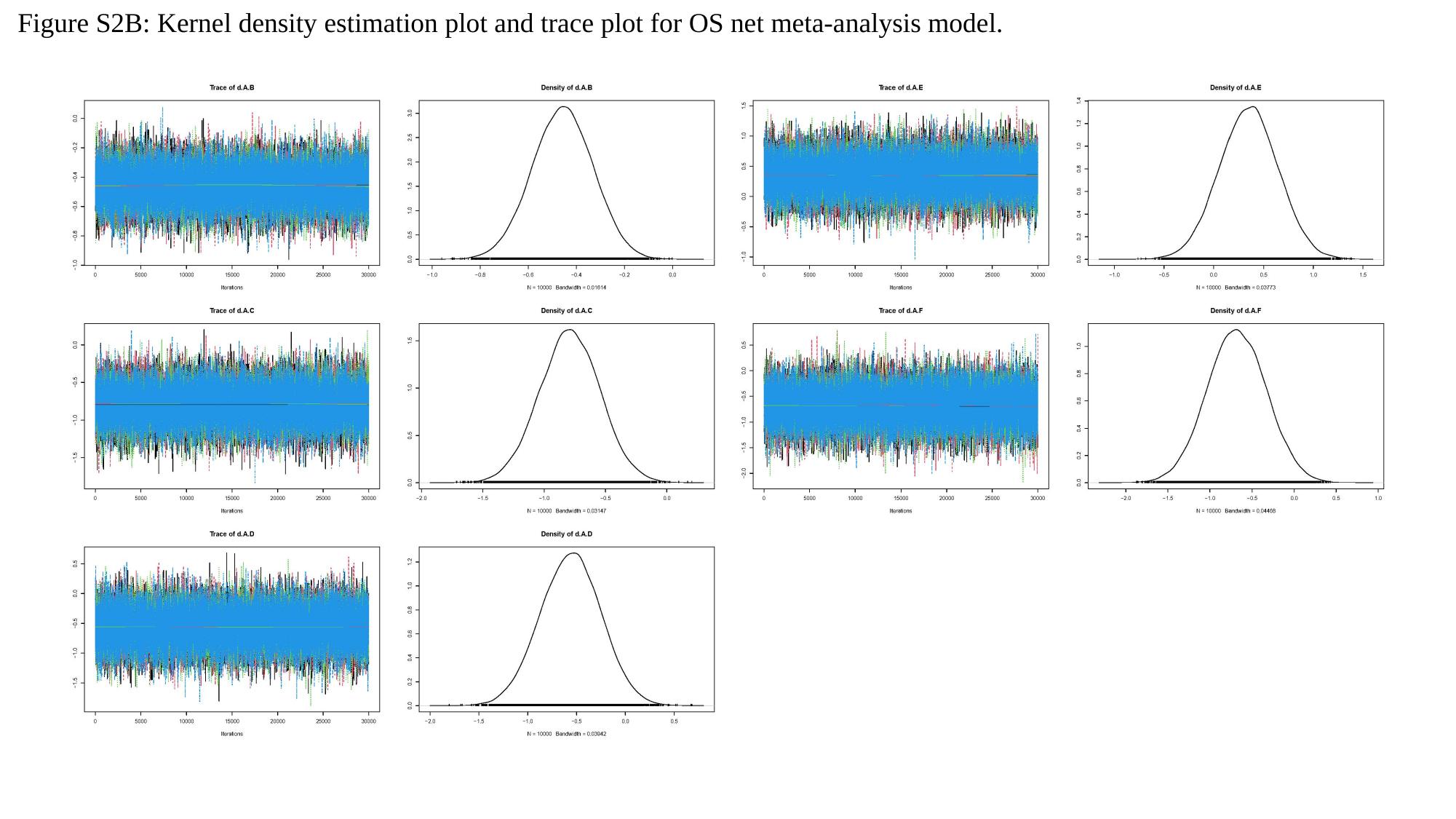

Figure S2B: Kernel density estimation plot and trace plot for OS net meta-analysis model.
